# Treatment of osteonecrosis of the jaw related to bisphosphonates and other antiresorptive agents

**DOI:** 10.4317/medoral.20980

**Published:** 2016-07-31

**Authors:** Francisco-Javier Rodriguez-Lozano, Ricardo-Elías Oñate-Sánchez

**Affiliations:** 1 DDS, PhD, Assistant Lecturer. Special Patients and Gerodontology Unit. School of Dentistry, University of Murcia, IMIB-Arrixaca, Spain; 2 MD, DDS, PhD, Senior Lecturer. Special Patients and Gerodontology Unit. School of Dentistry, University of Murcia, IMIB-Arrixaca, Spain

## Abstract

**Background:**

The clinical management of medication-related osteonecrosis of the jaw (MRONJ) in patients treated with bisphosphonates and other antiresorptive agents is subject to controversy. The American Association of Oral and Maxillofacial Surgeons (AAOMS) has developed guidelines for the correct management of the disorder which are revised and updated by a panel of experts.

**Material and Methods:**

The present systematic review analyzes the different treatments currently used to treat this clinical condition, based on the PRISMA® (Preferred Reporting Items for Systematic Reviews and Meta-Analyses) statement published in 2009. An electronic Medline search was made of the PubMed database, covering the period 2006-2014. The last search date was 31 December 2014.

**Results:**

A total of 29 articles were selected from the initial search according to the different drugs implicated in the appearance of osteonecrosis; the treatment modality used according to the stage of the disease; and the recorded success rate.

**Conclusions:**

It is currently still recommended that the management of MRONJ should be decided according to the stage of the disease – conservative treatment being preferred in early stages without symptoms, while surgical management is preferred in the case of bone exposure with symptoms.

**Key words:**Osteonecrosis, medication, bisphosphonates, treatments, review

## Introduction

The American Association of Oral and Maxillofacial Surgeons (AAOMS) has recently suggested changing the term “bisphosphonate-related osteonecrosis of the jaw (BRONJ)” to “medication-related osteonecrosis of the jaw (MRONJ)”, in view of the increase in frequency of maxillary and mandibular osteonecrosis related not only to bisphosphonates (BPs) but also to other antiresorptive agents such as denosumab and antiangiogenic drugs (1). The AAOMS position document has also modified the definition of MRONJ established in 2009 (2), and patients susceptible to MRONJ are now defined as those presenting the following characteristics: a) a history of antiresorptive or antiangiogenic drug treatment; b) the presence of bone exposure or intra- or extraoral fistulization for over 8 weeks without remission; and c) no history of radiotherapy or diseases metastasizing to the maxilla.

However, osteonecrosis of the jaw associated to these drugs has been the subject of controversy ever since the first case was reported in 2003 (3). The AAOMS considers prevention to be the key element in dealing with MRONJ. In this regard, a multidisciplinary team in which the dentist plays a fundamental role is required to define management of the lesions. Consultation of the dentist before starting antiresorptive or antiangiogenic drug treatment considerably reduces the risk of developing MRONJ in the event of osteonecrosis-triggering interventions such as tooth extractions (4-6).

Many authors have studied the management of MRONJ, including different treatments such as surgical debridement, resection of the lesions, oxygen therapy and recently also the use of mesenchymal cells to regenerate the damaged bone (7-9). The type of treatment depends on the diagnosis and clinical stage of MRONJ. A staging system was developed in 2006 by Ruggiero *et al.* (10), and was subsequently adopted by the AAOMS with updates in 2007, 2009 and 2014 (1,2,11).

Independently of the stage of the disease, initial treatment must seek to control the pain and bone infection / necrosis, in order to preserve patient quality of life (1).

Despite the current recommendations of the AAOMS advocating conservative management of MRONJ, a number of studies have found this approach to be successful in only 20% of the cases, versus in over 85% when surgical treatment is decided (12).

The present study offers a systematic review of the different treatments currently used in application to MRONJ reflected in the literature, based on the stage of the disease, and examines the results of each treatment modality according to the associated success rates.

## Material and Methods

The present systematic review was based on the PRISMA® (Preferred Reporting Items for Systematic Reviews and Meta-Analyses) statement published in 2009 (13). An electronic Medline search was made of the PubMed database, covering the period 2006-2014. The last search date was 31 December 2014. The identified references were processed using Endnote®.

Framing the question (PICO): What are the treatments used for medication-related osteonecrosis of the jaw and their success rates?

- Search strategy:

The literature search was made using the following key words: “medication”, “bisphosphonates”, “antiresorptive”, “antiangiogenic”, “denosumab”, “sunitinib”, “osteonecrosis”, “jaw” and “treatment”, combined with the boolean operators AND / OR. The electronic search was complemented by a manual search of the references found in the manuscripts and involving the following journals: *British Journal of Oral and Maxillofacial Surgery, International Journal of Oral and Maxillofacial Surgery, Journal of Oral and Maxillofacial Surgery, Oral Oncology, Oral Surgery, Oral Medicine, Oral Pathology, Oral Radiology and Endodontology, Journal of Periodontology, Medicina Oral, Patología Oral y Cirugía Bucal*. After eliminating duplications, the potential titles and abstracts were filtered based on the following criteria:

- Inclusion criteria:

Articles entirely in English, referred to medication-related osteonecrosis of the jaw (bisphosphonates, antiresorptive agents and antiangiogenic drugs) in humans, and without sample limitations.

- Exclusion criteria:

Publications in languages other than English, systematic reviews, experimental studies in animals, and letters to the Editor.

All the information was compiled from the selected articles by a single reviewer (FJRL). The documented variables included general data such as the author, country, year of publication, objectives, patient characteristics, and information referred to interventions and follow-up. The specific study variables included clinical response indicators such as disappearance of the lesions, recurrence or evolution of the disease, and the frequency and severity of adverse reactions for the assessment of safety.

## Results and Discussion

The systematic PubMed search adopting the search strategy: (medication) AND osteonecrosis) AND jaw) AND treatment) AND (“2006/01/01” [Date - Publication] : “2014/12/31” [Date - Publication]) yielded a total of 85 articles. After reading the title and abstract, 74 of the articles that failed to meet the inclusion criteria were excluded from the study. The search strategy: (bisphosphonates) AND osteonecrosis) AND treatment) AND (“2006/01/01” [Date - Publication]: “2014/12/31” [Date - Publication]) AND jaw yielded 1151 articles, of which 15 meeting the inclusion criteria were included in the study. Lastly, the search strategy: (denosumab) AND osteonecrosis) AND treatment) AND (“2006/01/01” [Date-Publication]: “2014/12/31” [Date-Publication]) AND jaw yielded 74 articles, of which three meeting the inclusion criteria were included in the study.

**Figure 1:**
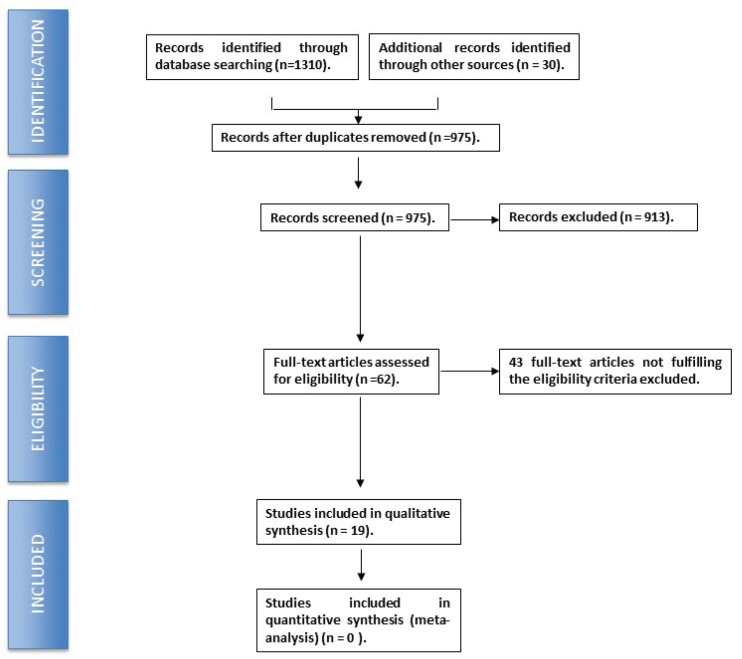
PRISMA® flowchart describing the search strategy and inclusion of the studied articles.

Following the electronic search and selection of the publications that met the established inclusion criteria, a total of 29 articles were selected (Fig. 1). A brief description of these studies is provided below, together with a summary of the different treatment options currently used in application to medication-related osteonecrosis of the jaw (MRONJ).

a) Conservative management

Patients amenable to conservative management would be those considered to be at risk and/or individuals without symptoms (stage I) (1). We could also include patients who for health reasons are not candidates for surgical treatment, or who are receiving cancer treatment. Due to the intrinsic definition of MRONJ, which implies “exposed bone”, surgery in principle would be the treatment option of choice - though some authors consider that conservative management can improve or keep the disease asymptomatic in up to 70% of the cases. Nevertheless, this favorable percentage cannot be regarded as representing treatment success, though temporary patient relief is provided (14). Conservative management includes the reinforcement of oral hygiene, periodic dental checks, oral rinses with chlorhexidine, and antibiotic treatment. In this regard, the most widely used antibiotics are amoxicillin with or without clavulanic acid (500 mg/1 g) clindamycin (300 mg), azithromycin (500 mg) and in some cases the combination of metronidazole with betalactams. In most of the studies, this approach resulted in the stabilization of osteonecrosis or simply the improvement of symptoms (14,15). In fact, higher success rates are only achieved when such treatments are combined with other conservative measures such as ozone therapy, hyperbaric oxygen and low-power laser therapy - though the rates are not comparable to those obtained with surgery (16).

Ozone therapy stimulates cell proliferation and soft tissue healing, and reduces pain, with promising results in phase I/II clinical trials. As such, this treatment modality could constitute a new alternative in the management of MRONJ (17). Hyperbaric oxygen (HBO) has sometimes been used in application to MRONJ, with controversial results. Historically, the healing action of HBO has been attributed to the creation of beneficial oxygen gradients. The reason for using HBO in MRONJ is that different authors consider it to improve wound healing, reduce edema and swelling, stimulate stem cell mobilization, and moderate the bone turnover suppression caused by bisphosphonates (16,18). Low-intensity laser therapy (LILT) has been shown to be an innovating and effective treatment in medicine, with effects that include the lessening of pain, improved wound healing, and the facilitation of nerve regeneration. It also exerts antimicrobial effects and facilitates the healing of wounds in the oral cavity, including the stimulation of reepithelization after periodontal or third molar surgery. Different studies have warranted the use of LILT on the basis of its biostimulating effects in MRONJ lesions (16,19).

Pentoxifylline and α-tocopherol have been suggested to assist antimicrobial therapy in the early stages of MRONJ, since these substances have been found to reduce the bone exposure area and symptoms in 74% of the cases (16,20).  Table 1 summarizes the studies made using conservative treatments in MRONJ, with the associated success rates (21-23).

**Table 1:**
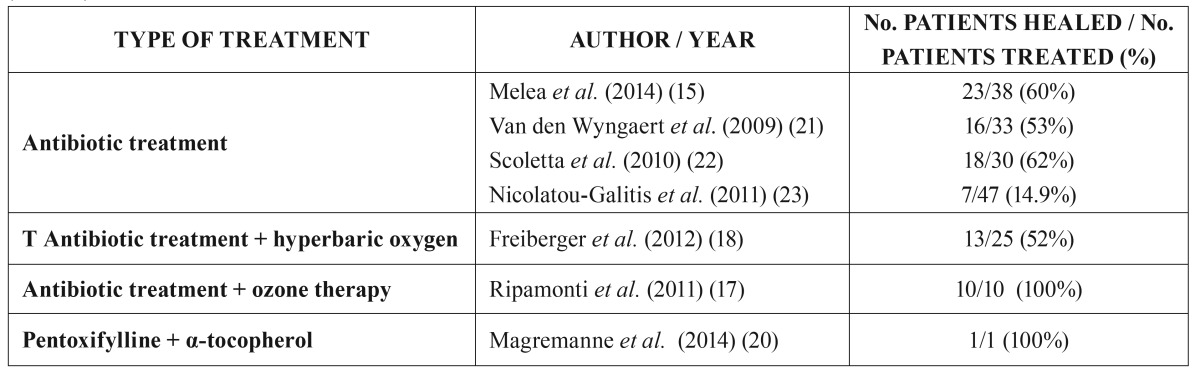
Summary of studies involving a conservative approach to the management of medication-related osteonecrosis of the jaw. (MRONJ).

a) Surgical management, alone or in combination with other treatments

There is general agreement on the advisability of surgery in those cases characterized by chronic exposure of necrotic bone, since the latter can interfere with wound healing and is of course infected (24). In these cases, minimum necessary necrotic bone elimination is indicated, and two surgical approaches have been recommended in this respect: (i) conservative debridement or surgery; and (ii) segmental resection. This latter approach applies to stage II/III MRONJ and cases in which nonsurgical conservative management has failed (24) ( Table 2).

**Table 2:**
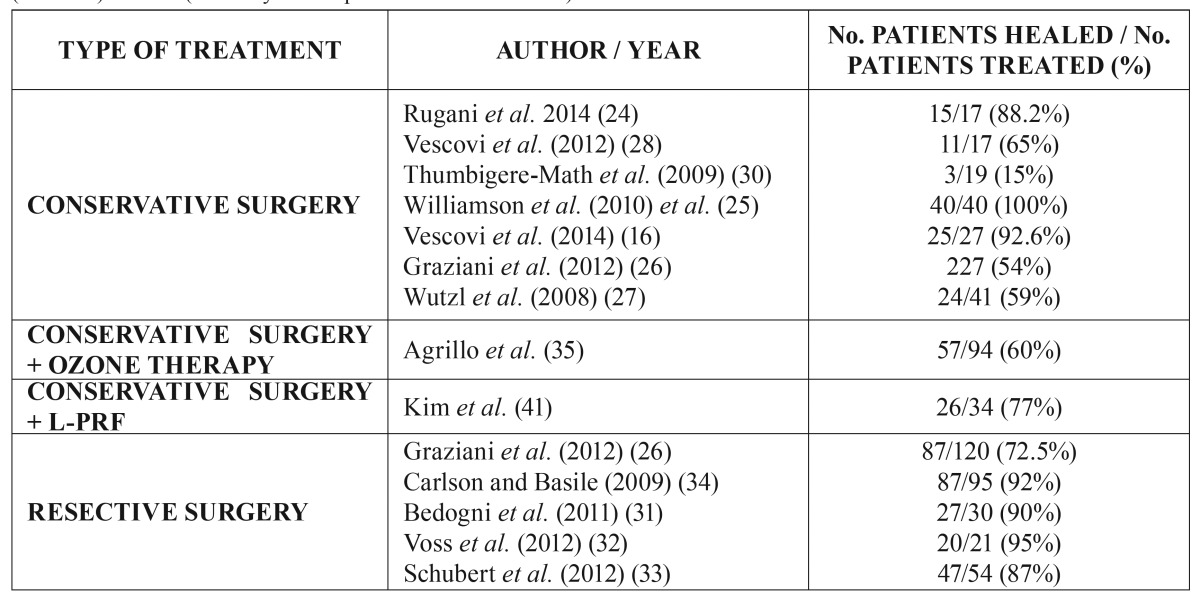
Summary of studies involving a surgical approach to the management of medication-related osteonecrosis of the jaw (MRONJ). L-PRF (Leukocyte- and platelet-rich fibrin mesh).

i.) Conservative surgery

Conservative surgery involves the removal of dead bone (sequestrectomy) and/or superficial surgical debridement of necrotic bone associated to oral antibiotics and chlorhexidine rinses. Most authors recommend scantly invasive surgery for MRONJ, since according to the reviewed literature this approach affords healing rates of over 50% (16,25-27). Wutzl *et al.* (27) carried out the first prospective study evaluating the outcome of surgery after 6 months in a cohort of 58 patients with osteonecrosis of the jaw. They found 58.5% of the patients to be free of pain and with an intact oral mucosa. Eleven out of 12 patients subjected to flap procedures for soft tissue closure presented a healthy mucosa. The authors showed that minimal resection of the necrotic bone and local soft tissue closure can afford satisfactory results.

Complementary treatments added to surgery have also been described in the literature. Vescovi *et al.* (28) obtained good results in MRONJ by combining surgical debridement with laser therapy. However, Atalay *et al.* (19) observed no statistically significant benefits with this approach compared with conventional surgery. Martins *et al.* (29) conducted a study in patients treated with antibiotics plus surgery, followed by low-intensity laser therapy and platelet-rich plasma applied to the surgical wound. The healing rates were found to be higher than in the patients subjected only to surgery and antibiotic treatment. In contrast, other investigators such as Thumbigere-Math *et al.* recorded a success rate of only 15% and observed colonization by Actinomyces spp. in those cases with exposed bone (30).

ii) Resective or extensive (segmental) surgery

In those patients in which previous treatment has failed, or in very advanced cases of MRONJ, resective or extensive (segmental) surgery is indicated with the purpose of eliminating all the necrotic tissue, leaving only healthy bone. However, resective surgery has generated controversy, since in many cases it is difficult to eliminate all the necrotic bone and guarantee the obtainment of healthy bone margins (31-33). Carlson and Basile (34) reported a very high success rate (92%) in MRONJ patients subjected to mandibular segmental resection and partial maxillectomy with the purpose of securing clean margins with healthy bone. However, disinfection measures associated to the surgical procedure are needed - hence the combination of surgery with other therapies such as ozone (35), laser irradiation (16) and, in particular, prolonged antibiotic treatment (generally penicillin, or tetracycline or clindamycin in patients allergic to penicillin) (36). Additional measures such as the use of stem cells (9,37), platelet-rich plasma (38), the administration of parathyroid hormone (39,40), or the use of leukocyte and platelet-rich fibrin meshes (41) are also promising strategies - though further clinical studies are needed in order to confirm their efficacy / effectiveness.

## Conclusions

It is currently still recommended that the management of MRONJ should be decided according to the stage of the disease – conservative treatment being preferred in early stages without symptoms, while surgical management is preferred in the case of bone exposure with symptoms. Effective and scantly invasive alternatives affording good patient quality of life will be the future treatments of choice, though at present there is no agreement as to which management approach is best suited to each specific circumstance.
